# Correlation analysis of serum intestinal fatty acid binding protein, D-lactic acid and intercellular adhesion factor-1 in sepsis patients with intestinal ischemia

**DOI:** 10.5937/jomb0-61181

**Published:** 2026-04-15

**Authors:** Yujie Shu, Bo Lao, Zhangzhang Song, Zhixing Zhuang, Hongzhang Li

**Affiliations:** 1 Yinzhou District Second Hospital, Department of Gastroenterology, Ningbo City, Zhejiang Province, China; 2 The First Affiliated Hospital of Fujian Medical University, Laboratory Department, Fuzhou City, China

**Keywords:** intestinal ischemia with sepsis, serum intestinal fatty acid binding protein, D-lactic acid, intercellular adhesion factor-1, prognostic correlation, intestinalna ishemija kod sepse, serumski intestinalni protein za vezivanje masnih kiselina, D-laktatna kiselina, međućelijski faktor adhezije-1, prognostička korelacija

## Abstract

**Background:**

To analyse the correlations between the levels of serum intestinal fatty acid binding protein (I-FABP), D-lactic acid (D-Lac), and intercellular adhesion factor-1 (ICAM-1) in patients with sepsis complicated with intestinal ischemia-reperfusion injury and the degree of intestinal mucosal injury and patient prognosis.

**Methods:**

A total of 236 patients with sepsis complicated with intestinal ischemia-reperfusion injury were included in the reperfusion injury group, and 176 patients with sepsis alone during the same period were included in the sepsis group. Based on the extent of intestinal mucosal damage, individuals in the reperfusion injury group were split into three groups: mild injury (52 patients), moderate injury (116 patients), and severe injury (68 patients). The patients were further divided into a good prognosis group (188 patients) and a poor prognosis group (48 patients). The general clinical data of all patients, including laboratory test indicators, were collected and grouped for comparison before and after treatment. Correlation analysis was conducted via Spearman correlation analysis. Predictive value assessment was conducted via receiver operating characteristic (ROC) curves.

**Results:**

The reperfusion damage group had higher levels of I-FABP D-Lac, ICAM-1, and IMA than the sepsis alone group, whereas the GLP-1 level was lower (P&lt; 0.05) than the sepsis alone group. Before treatment, the levels of I-FABP D-Lac, ICAM-1, and IMA in the mild, moderate, and severe injury groups increased successively, and the level of GLP-1 decreased successively (P&lt; 0.05). After treatment, the levels of I-FABP D-Lac, ICAM-1, and IMA in the three groups of patients were all lower than those before treatment in the same group, and the levels of the above indicators in the severe injury group were all greater than those in the mild and moderate injury groups during the same period. The GLP-1 levels of the three patient groups were all higher than those before treatment within the same group. In comparison to the mild and moderate damage groups, the severe injury group's GLP-1 levels were lower during the same time period (P&lt; 0.05). Spearman correlation analysis revealed that the levels of I-FABP D-Lac, ICAM-1, and IMA were positively correlated with the AGI grade. In contrast, the level of GLP-1 was negatively correlated with the AGI grade (P&lt; 0.05). While the level of GLP-1 was lower than that of the good prognosis group (P&lt; 0.05), the levels of I-FABP D-Lac, ICAM-1, and IMA were higher in the bad prognosis group. The ROC curve analysis revealed that the combined predictive value of I-FABP D-Lac, ICAM-1, IMA, and GLP-1 for poor prognosis in patients with sepsis complicated by intestinal ischemia-reperfusion injury was greater than that of these five indicators alone (P&lt; 0.05).

**Conclusions:**

The levels of serum I-FABP, D-Lac, ICAM-1, IMA, and GLP-1 in patients with sepsis complicated with intestinal ischemia-reperfusion injury are closely related to the degree of intestinal mucosal injury and prognosis. The combined prediction of short-term prognosis has high clinical value.

## Abstract (sr)

**Uvod:**

Cilj je bio analiza korelacije između nivoa serumskog intestinalnog proteina za vezivanje masnih kiselina (I-FABP), D-laktatne kiseline (D-Lac) i međućelijskog faktora adhezije-1 (ICAM-1) kod pacijenata sa sepsom komplikovanom intestinalnom ishemijsko-reperfuzionom povredom, stepena oštećenja crevne mukoze i prognoze pacijenata.

**Metode:**

Ukupno 236 pacijenata sa sepsom komplikovanom intestinalnom ishemijsko-reperfuzionom povredom je uključeno u grupu reperfuzione povrede, dok je 176 pacijenata samo sa sepsom u istom periodu uključeno u grupu sepse. Na osnovu stepena oštećenja crevne mukoze, pacijenti iz grupe reperfuzione povrede su podeljeni u tri podgrupe: blago oštećenje (52 pacijenta), umereno oštećenje (116 pacijenata) i teško oštećenje (68 pacijenata). Pacijenti su dalje podeljeni u grupu sa dobrom prognozom (188 pacijenata) i grupu sa lošom prognozom (48 pacijenata). Prikupljeni su opšti klinički podaci svih pacijenata, uključujući laboratorijske parametre, i poređeni pre i posle tretmana. Korelaciona analiza je sprovedena Spearmanovom korelacionom analizom. Procena prediktivne vrednosti je sprovedena pomoću ROC krivih.

**Rezultati:**

Grupa sa reperfuzionim oštećenjem je imala više nivoe I-FABP D-Lac, ICAM-1 i IMA u poređenju sa grupom samo sa sepsom, dok je nivo GLP-1 bio niži (P&lt; 0,05). Pre tretmana, nivoi I-FABP D-Lac, ICAM-1 i IMA u grupama sa blagim, umerenim i teškim oštećenjem su rasli sukcesivno, dok je nivo GLP-1 sukcesivno opadao (P&lt; 0,05). Nakon tretmana, nivoi I-FABP D-Lac, ICAM-1 i IMA u sve tri grupe pacijenata bili su niži nego pre tretmana, dok su nivoi pomenutih parametara u grupi sa teškim oštećenjem bili viši u poređenju sa grupama sa blagim i umerenim oštećenjem u istom periodu. Nivoi GLP-1 u sve tri grupe su bili viši nego pre tretmana, dok su nivoi GLP-1 u grupi sa teškim oštećenjem bili niži u poređenju sa grupama sa blagim i umerenim oštećenjem (P&lt; 0,05). Spearmanova analiza je pokazala da su nivoi I-FABP, D-Lac, ICAM-1 i IMA pozitivno korelisali sa stepenom AGI, dok je nivo GLP-1 bio negativno povezan sa stepenom AGI (P&lt; 0,05). U poređenju sa grupom sa dobrom prognozom, grupa sa lošom prognozom je imala više nivoe I-FABP D-Lac, ICAM-1 i IMA, dok je nivo GLP-1 bio niži (P&lt; 0,05). Analiza ROC krive je pokazala da kombinovana prediktivna vrednost I-FABP D-Lac, ICAM-1, IMA i GLP-1 za lošu prognozu kod pacijenata sa sepsom i intestinalnom ishemijsko-reperfuzionom povredom ima veću vrednost nego bilo koji od ovih parametara pojedinačno (P&lt; 0,05).

**Zaključak:**

Nivoi serumskog I-FABP, D-Lac, ICAM-1, IMA i GLP-1 kod pacijenata sa sepsom komplikovanom intestinalnom ishemijsko-reperfuzionom povredom usko su povezani sa stepenom oštećenja crevne mukoze i prognozom. Kombinovana analiza ima visok klinički značaj u davanju kratkoročne prognoze.

## Introduction

Sepsis is a complex disease with a relatively high mortality rate [1]. The intestinal mucosa is a crucial biological barrier in the human body. However, patients with sepsis often have varying degrees of intestinal mucosal damage, which is not conducive to a good prognosis [2][3][4]. If human intestinal cells remain in a state of ischemia for a prolonged period, the sudden restoration of blood supply can induce intestinal ischemia-reperfusion injury. Intestinal ischemia-reperfusion injury can compromise intestinal barrier function, stimulate the release of inflammatory factors, and exacerbate the condition [5]. Thus, early evaluation of the intestinal mucosal state in patients with sepsis accompanied by intestinal ischemia-reperfusion injury, along with the application of preventive and therapeutic measures, has emerged as a critical clinical issue. Serum intestinal fatty acid binding protein (I-FABP) is a specific small-molecule cytoplasmic protein, and D-lactic acid (D-Lac) is a chemical substance produced by inherent bacteria in the intestine. When there is ischemia or injury in the intestine, the levels of both enzymes increase significantly [6]. Intercellular adhesion factor (ICAM)-1 has been proven to be closely related to lung injury and is also a sensitive marker of tissue damage [7]. However, it remains unknown whether these three factors can be combined to predict intestinal ischemia-reperfusion injury in patients with sepsis.

A potentially fatal organ malfunction brought on by the host's dysregulated reaction to infection, called sepsis [8][9][10]. A high incidence and mortality rate characterise it, and it is a significant public health challenge worldwide. As the largest immune organ and a vital barrier organ in the human body, the intestine plays dual roles as both an »engine« and a »target organ« in the pathophysiological process of sepsis. Intestinal ischemia is a common and serious complication in patients with sepsis. It occurs due to hemodynamic disorders, microcirculation disorders, and coagulation activation caused by systemic inflammatory response syndrome (SIRS), resulting in a reduced blood supply to the intestinal mucosa. Intestinal ischemia can lead to damage, apoptosis, and even necrosis of intestinal epithelial cells, thereby destroying the integrity of the intestinal mucosal barrier [11]. Early identification of whether patients with sepsis have intestinal ischemia is crucial for timely intervention and improving prognosis. However, the commonly used diagnostic methods in clinical practice at present (such as imaging and clinical symptoms) often lack sufficient sensitivity and specificity or lag. Therefore, it is of great clinical value to search for serological biomarkers that can sensitively and specifically reflect early changes in intestinal ischemic injury in sepsis [12]. When intestinal epithelial cells are damaged, they can be rapidly released into the bloodstream and serve as sensitive indicators of damage to the intestinal epithelium. D-Lactic acid is produced mainly by the metabolism of gram-positive bacteria in the intestinal tract, such as Lactobacillus and Bifidobacterium, and is rarely absorbed under normal circumstances. When the intestinal barrier is damaged and its permeability increases, D-lactic acid can pass through the damaged intestinal wall and enter the bloodstream, serving as a marker for assessing intestinal barrier permeability and the risk of bacterial transmigration [13]. Vascular endothelial cells and other immune cells exhibit intercellular adhesion molecule-1 (ICAM-1), a crucial component that mediates inflammation. Under inflammatory stimulation, its expression is significantly upregulated, and it enters the circulatory system (sICAM-1), where it mediates white blood cell adhesion and migration, participates in inflammatory cascade amplification and endothelial cell damage, and is a key indicator for evaluating the systemic inflammatory response and endothelial dysfunction [14][15][16]. The value of detecting all three indicators together in the early diagnosis, disease assessment, and prognosis prediction of intestinal ischemia in sepsis patients should be assessed.

Additionally, all serum biomarker detection methods used in this study were conducted using established laboratory techniques. The concentration determination of I-FABP and ICAM-1 (including its soluble form sICAM-1) mainly uses enzyme-linked immunosorbent assay (ELISA), which is based on antigen antibody-specific reactions. It features high sensitivity, good specificity, and relatively simple operation [17]. The detection of D-lactic acid is usually carried out via enzymatic spectrophotometry (enzymatic spectrophotometric assay), which uses D-lactate dehydrogenase (D-LDH) to catalyse the oxidation of D-lactic acid specifically. Through coupling reactions, products that spectrophotometers can detect are generated. Accurate quantification of its concentration can be achieved. These mature serological detection techniques provide a reliable methodological basis for smooth implementation [18][19][20].

Our study included 236 patients with sepsis complicated by intestinal ischemia-reperfusion injury, aiming to analyse the correlations between the levels of I-FABP D-Lac, and ICAM-1 and the degree of intestinal mucosal injury and prognosis.

## Materials and methods

### Included research data

A total of 236 patients with sepsis complicated by intestinal ischemia-reperfusion injury were admitted to our hospital from January 2023 to January 2025 (reperfusion injury group). The patients had an average age of 48.04±6.09 years, comprising 120 females and 116 males, with ages ranging from 20 to 77 years.

Inclusion criteria: (1) Met the diagnostic criteria for acute gastrointestinal dysfunction in sepsis patients; (2) Met the diagnostic criteria for intestinal ischemia-reperfusion injury. Exclusion criteria: (1) Had acute gastrointestinal disease within the previous month; (2) Had a previous history of gastrointestinal surgery; (3) Had chronic hepatitis B, myocarditis, diabetes or other diseases that may affect the levels of I-FABP D-Lac and ICAM-1; (4) Had a malignant tumor; (5) Died from a disease other than sepsis or intestinal ischemia.

The sepsis group consisted of 176 patients with sepsis alone who were admitted to our hospital during the same time period. There were 92 men and 84 females, aged 21-75, with an average age of 47.87±6.05 years. In the reperfusion injury group, patients were categorised based on the extent of intestinal mucosal damage into three groups: mild injury (52 patients), moderate injury (116 patients), and severe injury (68 patients). This study was approved by the hospital ethics committee (No. HKYS-2025-A0169), and all patients provided informed consent.

### Collection of laboratory inspection indicators

The data included sex, age, infection focus, and the levels of I-FABP D-Lac, ICAM-1, human glucagon-like peptide 1 (GLP-1), and human ischemia-modified albumin (IMA) on the day of admission and 3 days after receiving relevant standardised treatments (intestinal drug support therapy, antioxidant free radical therapy, etc.) in accordance with guideline standards. The levels of I-FABF, D-Lac, GLP-1, and ICAM-1 were detected using an enzyme-linked immunosorbent assay, and the level of IMA was determined using a colourimetric method.

### Assessment of the degree of intestinal function impairment

All patients underwent assessment of the degree of intestinal mucosal injury after admission, referring to the standards in the »Clinical Expert Consensus for Acute Gastrointestinal Dysfunction in Sepsis«. Partial impairment of gastrointestinal function is classified as mild injury »acute gastrointestinal injury (AGI) grade I«, gastrointestinal insufficiency without affecting the overall condition is classified as moderate injury (AGI grade II), and gastrointestinal failure with no improvement in systemic symptoms/gastrointestinal failure with severe impact on the functions of other organs is classified as severe injury (AGI grade III/AGI) (level IV).

### Prognostic follow-up

Following discharge, all patients received a three-month phone follow-up, and the short-term prognosis was noted. If the discharge outcome was death within 3 months, the patient was judged as having a poor prognosis. Patients in the reperfusion injury group were further divided into a good prognosis group (188 patients) and a poor prognosis group (48 patients).

### Statistical processing

Statistical analysis was conducted via SPSS 23.0 software. Expressed as x̄±s, the measurement results follow a normal distribution. Analysis of variance in one direction was employed for comparisons among several groups, and a t-test was used for comparisons between two groups. Count data are expressed as the number of cases and percentages, and the χ^2^ test was used for comparisons between groups. Correlation analysis was conducted via Spearman correlation analysis. Predictive value assessment was conducted via receiver operating characteristic (ROC) curves. A P value <0.05 was considered to indicate statistical significance.

## Results

### Comparison of general clinical data between patients in the sepsis alone group and those in the reperfusion injury group

The two patient groups did not differ statistically significantly in terms of age, sex, or infection foci (P>0.05) (Table 1). Compared with patients with uncomplicated sepsis, patients with sepsis complicated with mesenteric ischemia-reperfusion injury have clinical characteristics such as older age, greater burden of underlying cardiovascular and metabolic diseases, more critical conditions (higher SOFA/APACHE II score), a greater proportion of abdominal cavity infection, greater need for organ support (higher mechanical ventilation rate), and poorer prognosis (significantly increased 28-day mortality). In addition to sex distribution, statistically significant differences were observed in multiple baseline indicators (P<0.05) between the two groups.

**Table 1:** Comparison of general clinical data of patients in the reperfusion group and the sepsis alone group [Cases (%)].

Group	Number<br>of cases	Gender		Age (years,<br>x̄±s)	Infection focus			
		Male	Female		Urinary<br>tract	Lung	Abdominal<br>cavity	Others
Sepsis alone group	176	91 (52.27)	85 (47.73)	47.87±6.05	35 (20.45)	91 (52.27)	19 (11.36)	31 (15.91)
Reperfusion injury<br>group	236	115 (49.15)	121 (50.85)	48.04±6.09	94 (40.68)	78 (33.90)	25 (11.86)	39 (13.56)
x^2^/t value		0.098		0.141		5.357		
P value		0.754		0.889		0.147		

### Comparison of the serum levels of I-FABP, D-Lac, ICAM-1, IMA and GLP-1 between the sepsis alone group and the reperfusion injury group

While the level of GLP-1 was lower than that of the sepsis alone group (P<0.05), the levels of I-FABP D-Lac, ICAM-1, and IMA were higher in the reperfusion damage group than in the sepsis alone group (Table 2).

**Table 2:** Comparison of serum Levels of I-FABP D-Lac, ICAM-, IMA, and GLP- between patients with sepsis alone and those with reperfusion injury (x̄ ± s).

Group	Number of cases	I-FABP (mg/L)	D-Lac (mg/L)	ICAM-1 (μg/L)	IMA (U/mL)	GLP-1 (pmol/L)
Sepsis alone<br>group	176	754.39±134.50	8.79±1.63	189.48±29.46	50.73±7.40	14.69±2.16
Reperfusion<br>injury group	236	1049.26±148.27	16.02±2.18	276.94±32.70	64.59±8.15	10.30±1.64
t value		11.946	18.474	14.001	8.876	11.729
P value		<0.001	<0.001	<0.001	<0.001	<0.001

The detection and analysis of serum markers in 176 patients with uncomplicated sepsis (sepsis group) and 236 patients with sepsis combined with intestinal ischemia-reperfusion injury (reperfusion injury group) revealed significant differences in each index between the two groups (P<0.05). Serum levels of D-lactic acid (D-Lac) and intestinal fatty acid binding protein (I-FABP), intercellular adhesion factor-1 (ICAM-1), and ischemia-modified albumin (IMA) in the reperfusion injury group were significantly greater than those in the sepsis group, while the sepsis group's level of glucagon-like peptide-1 (GLP-1) was noticeably higher. Patients with sepsis complicated with intestinal ischemia reperfusion injury have more severe intestinal epithelial cell injury (I-FABP), intestinal barrier permeability disruption and bacterial translocation risk (D-Lac), systemic inflammatory response activation (ICAM-1), and a tissue ischemia-hypoxia state. Moreover, it is accompanied by significant weakening of intestinal self-protection and repair functions (GLP-1). The serological characteristics of these synergistic changes provide crucial laboratory evidence for identifying intestinal ischemia-reperfusion injury in patients with sepsis, highlighting the complexity of the pathophysiological state and the increased clinical risk in such patients.

### Comparison of general clinical data among patients in the mild injury group, moderate injury group and severe injury group

There were no statistically significant differences in sex, age, or infection foci among the three patient groups (P>0.05) (Table 3). Patients with varying degrees of damage exhibit notable variations in their clinical presentations. The age and sex ratios, as well as the incidence of comorbidities and underlying diseases, of patients in the mild injury group were relatively low. However, the incidence of age, comorbidities, and underlying disorders was higher among individuals in the severe damage group. This finding suggests that as the degree of injury increases, so does the overall health status of patients and the complexity of their underlying diseases. In addition, the disease course of patients in the severe injury group was longer. The degree of abnormalities in physiological indicators and laboratory tests at admission was significantly greater than that in the mild and moderate injury groups, further reflecting that the condition of patients with severe injuries was more serious.

**Table 3:** Comparison of data of patients in the mild injury group, moderate injury group and severe injury group [Cases (%)].

Group	Cases	Gender		Age<br>(Years)	Infection focus			
		Male	Female		Urinary tract	Lung	Abdominal<br>cavity	Others
Mild injury group	52	23 (46.15)	29 (53.85)	47.73±6.08	12 (23.08)	24 (46.15)	3 (7.69)	13 (23.08)
Moderate injury<br>group	116	65 (51.72)	51 (48.28)	48.60±6.11	63 (55.17)	23 (20.69)	16 (13.79)	14 (10.34)
Severe injury<br>group	68	32 (47.06)	36 (52.94)	47.99±6.04	20 (29.41)	32 (47.06)	8 (11.76)	8 (11.76)
x^2^/t value		0.153	0.111	7.367				
P value	0.926	0.895	0.288					

### Comparison of I-FABP, D-Lac, ICAM-1, IMA and GLP-1 levels before and after treatment in patients with different degrees of intestinal mucosal injury

Before treatment, the levels of I-FABP D-Lac, ICAM-1 and IMA in patients in the mild, moderate and severe injury groups increased successively, and the level of GLP-1 decreased successively (P<0.05). After treatment, the levels of I-FABP D-Lac, ICAM-1 and IMA in the three groups of patients were all lower than those before treatment in the same group, and the levels of the above indicators in the severe injury group were all greater than those in the mild and moderate injury groups during the same period. All three patient groups had higher GLP-1 levels than they had before treatment. The severe damage group's GLP-1 levels were lower than those of the mild and moderate injury groups over the same time period (P<0.05) (Table 4).

**Table 4:** Comparison of I-FABP D-Lac, ICAM-, IMA, and GLP- levels before and after treatment in patients with different degrees of intestinal mucosal injury groups (x̄ ± s).

Group		Number<br>of cases	I-FABP<br>(mg/L)	D-Lac<br>(mg/L)	ICAM-1(μg/L)	IMA<br>(U/mL)	GLP-1<br>(pmol/L)
Mild injury<br>group	Before<br>treatment	52	891.72±105.49	10.81±1.22	226.97±31.05	58.76±8.02	16.24±2.86
	After<br>treatment	52	673.14±99.25	9.42±1.07	186.11±29.73	50.60±7.84	20.51±2.47
Moderate<br>injury group	Before<br>treatment	116	1180.35±117.61	16.73±3.80	284.18±36.51	65.22±8.36	14.60±2.77
	After<br>treatment	116	854.73±101.65	11.28±2.51	220.76±32.49	56.17±6.97	17.08±2.15
Severe<br>injury group	Before<br>treatment	68	1359.46±129.45	21.19±5.30	309.62±40.83	72.16±9.10	11.09±2.13
	After<br>treatment	68	927.43±105.84	13.66±2.79	250.81±31.74	63.03±7.01	15.70±2.08

### Correlation analysis of the serum levels of I-FABP D-Lac, ICAM-1, IMA, GLP-1 and AGI grade in patients with sepsis complicated with intestinal ischemia-reperfusion injury

Spearman correlation analysis revealed that in sepsis patients with intestinal ischemia-reperfusion injury, the levels of serum I-FABP D-Lac, ICAM-1 and IMA were significantly positively correlated with the grade of AGI (acute gastrointestinal injury). However, the level of GLP-1 was significantly negatively correlated with the AGI grade. This means that as the AGI grade increases, i.e., as the degree of intestinal injury worsens, the levels of I-FABP D-Lac, ICAM-1, and IMA in patients also increase accordingly, whereas the level of GLP-1 decreases accordingly. This result strongly supports the potential value of I-FABP, D-Lac, ICAM-1 and IMA as markers of intestinal injury. The increase in their levels may reflect the disruption of the intestinal barrier and the activation of the inflammatory response. Moreover, the decrease in GLP-1 levels may indicate impaired intestinal endocrine function.

### Comparison of the levels of I-FABP, D-Lac, ICAM-1, IMA and GLP-1 between the poor prognosis group and the good prognosis group

The levels of I-FABP, D-Lac, ICAM-1 and IMA in the poor prognosis group were greater than those in the good prognosis group, whereas the level of GLP-1 was lower than that in the good prognosis group (P<0.05) (Table 5).

**Table 5:** Comparison of IFABP D-Lac, ICAM-4, IMA and GLP- levels (x̄ ± s).

Group	Number of<br>cases	I-FABP (mg/L)	D-Lac (mg/L)	ICAM-1 (μg/L)	IMA (U/mL)	GLP-1 (pmol/L)
Poor prognosis<br>group	48	1477.51±132.50	21.67±4.61	317.82±41.03	67.15±8.37	10.34±2.06
Good prognosis<br>group	188	1079.37±124.16	11.32±3.17	244.59±35.29	55.33±7.99	15.40±2.82
t value		9.784	9.158	6.209	4.404	5.816
P value		<0.001	<0.001	<0.001	<0.001	<0.001

In sepsis patients with intestinal ischemia-reperfusion injury, the poor prognosis group had significantly higher levels of I-FABP D-Lac, ICAM-1, and IMA than the good prognosis group, while the good prognosis group had significantly lower levels of GLP-1. These results suggest a strong correlation between a patient's poor prognosis and higher levels of I-FABP D-Lac, ICAM-1, and IMA, as well as lower levels of GLP-1. The differences in these indicators may reflect the severity of intestinal injury, the intensity of the inflammatory response, and disorders of intestinal endocrine function. These factors jointly affect the overall prognosis of patients. Specifically, elevated levels of I-FABP D-Lac and ICAM-1, which are markers of intestinal injury and inflammation, may indicate impaired intestinal barrier function, bacterial translocation and an intensified systemic inflammatory response, thereby leading to a poor prognosis. GLP-1, an intestinal hormone, has functions such as protecting intestinal function and regulating inflammatory responses. A decrease in its level may weaken the body's protective mechanism against intestinal damage, further aggravating the condition and leading to a poor prognosis.

### Predictive value of I-FABP D-Lac, ICAM-1, IMA and GLP-1 for poor prognosis in patients with sepsis complicated with intestinal ischemia-reperfusion injury

ROC curve analysis revealed that the combined predictive value of I-FABP D-Lac, ICAM-1, IMA, and GLP-1 for poor prognosis in patients with sepsis complicated by intestinal ischemia-reperfusion injury was greater than that of these five indicators alone (P<0.05) (Table 6).

**Table 6:** ROC curve analysis results of IFABP D-Lac, ICAM-4, IMA, and GLP for predicting poor prognosis in patients with sepsis complicated with intestinal ischemia-reperfusion injury.

Indicator	Best<br>Cutoff value	AUC	95%CI	P value	Yoden Number<br>of fingers	Sensitivity<br>(%)	Specificity<br>(%)
IFABP	307.16 mg/L	0.862	0.747~0.938	<0.001	0.663	83.33	82.98
D-L	18.73 mg/L	0.828	0.707~0.914	<0.001	0.559	75.00	80.85
ICAM-1	297.81 mg/L	0.706	0.573~0.817	<0.025	0.372	50.00	87.23
IMA	59.29 mg/L	0.832	0.712~0.916	<0.001	0.580	75.00	82.98
GLP-1	12.76 mg/L	0.892	0.784~0.958	<0.010	0.895	91.67	97.87
Five couplets<br>Combined<br>testing	-	0.975	0.897~0.998	<0.001	0.661	91.67	74.47

I-FABP D-Lac, ICAM-1, IMA and GLP-1 have potential value in predicting the poor prognosis of patients with sepsis accompanied by intestinal ischemia-reperfusion injury. More importantly, the predictive efficiency of jointly applying these five indicators for prediction is significantly greater than that of using any one of them alone. ROC curve analysis confirmed this finding, suggesting that the combined prediction model can more accurately distinguish between patients with good prognosis and those with poor prognosis. This may be because a single indicator can reflect only a specific aspect of the disease, whereas the combined prediction model integrates information from multiple aspects, such as intestinal injury, the inflammatory response, and intestinal endocrine function, thereby providing a more comprehensive reflection of the complex pathophysiological process of the disease.

## Discussion

Sepsis is considered a global health priority. It can cause systemic inflammatory responses in patients, leading to symptoms such as intestinal flora imbalance, mucosal ischemia and hypoxia [21][22][23]. If not treated in time, it can even cause multiple organ failure and shock, with a fatality rate as high as 50%. Currently, the pathogenesis of sepsis remains unclear. The theories of intestinal bacteria and endotoxin transfer are mainstream. Intestinal mucosal ischemic injury and sepsis are mutually causal, accelerating the progression of the disease. If diagnosis and treatment are not timely, it can increase the risk of adverse prognosis and endanger the patient's quality of life [24]. Currently, several studies have demonstrated that specific serum biomarkers can serve as indicators for the early assessment and diagnosis of sepsis complicated by acute ischemic injury. However, relevant research results on factors such as I-FABP D-Lac and ICAM-1 are somewhat limited. In clinical practice, the early assessment of intestinal mucosal injury often provides a timely disease assessment and early warning, guiding the prevention and treatment of sepsis and improving the prognosis of critically ill patients [25][26][27].

While the level of GLP-1 was lower than that of the sepsis alone group, the levels of I-FABP D-Lac, ICAM-1, and IMA were higher in the reperfusion damage group. I-FABP D-Lac, ICAM-1, and IMA levels increased in the mild, moderate, and severe damage groups prior to therapy, whereas GLP-1 levels progressively decreased. After treatment, the levels of I-FABP D-Lac, ICAM-1, and IMA in the three patient groups were lower than those before treatment in the same group. The levels of the above indicators in the severe injury group were greater than those in the mild injury group and the moderate injury group during the same period. All three patient groups had higher GLP-1 levels than they had before treatment. The GLP-1 level in the severe injury group was lower than that in the mild injury group and the moderate injury group during the same period. The levels of I-FABP D-Lac, ICAM-1 and IMA were positively correlated with the AGI grade, whereas the level of GLP-1 was negatively correlated with the AGI grade. These findings indicate that the levels of I-FABP D-Lac, ICAM-1, IMA, and GLP-1 are closely related to the degree of intestinal mucosal injury. As the disease progresses, I-FABP D-Lac, ICAM-1, and IMA are highly expressed, whereas GLP-1 is expressed at low levels.

The reason for this might be that the AGI grade assesses the degree of gastrointestinal injury from multiple dimensions, such as digestive tract symptoms and gastrointestinal motility disorders [28][29][30]. I-FABP is a protein unique to intestinal cells, and D-Lac is a common metabolic product of intestinal bacteria. Under normal circumstances, they rarely enter the bloodstream. However, Intestinal mucosal permeability increases in response to intestinal ulceration, and I-FABP and D-Lac are released into the bloodstream, resulting in an abnormal increase in their levels. ICAM-1 is a molecule involved in the intercellular mucosa that mediates the intercellular adhesion reaction. When the intestinal mucosa is damaged, ICAM-1 mediates the local aggregation and activation of neutrophils. The more severe the damage is, the greater the level of ICAM-1 [31]. IMA is often used in the diagnosis of myocardial ischemia, primarily due to its combination with albumin and disrupted amino sequences, as well as transition metals such as cobalt. However, this indicator is not limited to the heart but can also occur in the intestine. When the intestinal mucosa is damaged, it may be accompanied by the generation of free radicals and oxidative stress processes, thereby affecting the structure and function of serum ALB and subsequently increasing the level of IMA. GLP-1 is a polypeptide hormone produced in the intestinal tract and multiple other sites that promotes intestinal repair, inhibits bacteria and inflammation, and regulates intestinal function [32]. When the level of GLP-1 in the blood is low, its ability to repair the intestinal mucosa is limited, resulting in more severe damage. Therefore, in patients with sepsis combined with intestinal ischemia-reperfusion injury, pathological mechanisms such as increased intestinal mucosal permeability, enhanced neutrophil aggregation and oxidation reactions, and weakened cell repair ability contribute to the condition. However, reperfusion injury can effectively reduce the amount of adhesion factors in the blood perfusion area, control the inflammatory response, help alleviate damage to intestinal epithelial cells, promote the gradual repair of the intestinal mucosal barrier, improve the secretion function of L cells, and thereby help improve conditions [33]. Ultimately, it effectively reduces the levels of I-FABP D-Lac, ICAM-1 and IMA and increases the level of GLP-1.

The best predictor of a poor outcome for patients with sepsis exacerbated by intestinal ischemia-reperfusion injury is the combination of I-FABP D-Lac, ICAM-1, IMA, and GLP-1, indicating that the prognosis of patients with sepsis complicated by intestinal ischemia-reperfusion injury is not optimal [34]. I-FABP D-Lac, ICAM-1, IMA and GLP-1 may all be involved in the pathological process of intestinal mucosal injury, suggesting that it is necessary to detect these biological indicators in the early clinical stage. The possible reason for this is that high levels of I-FABP D-Lac, and ICAM-1, and low levels of IMA and GLP-1 can, to some extent, trigger systemic inflammatory responses and accelerate the progression of sepsis. It leads to serious consequences, such as septic shock and multiple organ failure, and ultimately results in a poor prognosis. In addition, patients with intestinal ischemia-reperfusion injury, owing to their more complex pathophysiological mechanisms, not only experience more severe digestive tract symptoms, such as diarrhoea and vomiting, but also have an increased risk of infection and multiple complications. During clinical testing, the level of I-FABP can be affected by factors such as rheumatoid factor and heterophilic antibodies. The D-Lac level can be affected by other lactic acid isomers or metabolite components in the blood [35]. The levels of ICAM-1 and GLP-1 are easily affected by factors such as immunosuppressants and anti-inflammatory drugs [36]. The IMA detection signal is susceptible to interference from certain nonspecific substances. Additionally, the levels of the above indicators are susceptible to factors such as sample stability, sample contamination, and detection methods [37][38][39]. However, joint detection can mitigate its weaknesses, maximise the avoidance of the influence of interfering factors, improve the accuracy of the results, and thereby achieve the highest predictive efficiency.

In conclusion, for sepsis patients who also have intestinal ischemia-reperfusion damage, the incidence of poor prognosis is relatively high. The prognosis of the patient and the extent of intestinal mucosal damage are strongly correlated with the levels of I-FABP D-Lac, ICAM-1, IMA, and GLP-1. The combined prediction of short-term prognosis has high application value. However, this study also has short-comings. The incidence of poor prognosis among patients is relatively high, which may be related to several factors. In the future, the sample size should be expanded and the universality of the results among patients improved to verify the research findings.

## Dodatak

### Conflict of interest statement

All the authors declare that they have no conflict of interest in this work.
